# Long-term migration of a cementless stem with different bioactive coatings. Data from a “prime” RSA study: lessons learned

**DOI:** 10.1080/17453674.2020.1840021

**Published:** 2020-11-04

**Authors:** Paul Van Der Voort, Martijn L D Klein Nulent, Edward R Valstar, Bart L Kaptein, Marta Fiocco, Rob G H H Nelissen

**Affiliations:** a Department of Orthopaedics, Leiden University Medical Center, Leiden;; b Department of Biomechanical Engineering, Faculty of Mechanical, Maritime and Materials Engineering, University of Technology Delft, Delft;; c Department of Medical Statistics and Bioinformatics, Leiden University Medical Center, Leiden;; d Mathematical Institute, Leiden University, Leiden, The Netherlands

## Abstract

Background and purpose — Little is known about the long-term migration pattern of cementless stems in total hip arthroplasty (THA). Furthermore, the role of bioactive coatings in fixation, and thus migration, remains uncertain. Hydroxyapatite (HA) is the most commonly used bioactive coating. However, delamination of the coating might induce loosening. Alternatively, fluorapatite (FA) has proved to be more thermostable than HA, thereby potentially increasing longevity. We assessed the long-term migration of cementless stems with different coatings using radiostereometric analysis (RSA), thereby establishing a reference for acceptable migration.

Patients and methods — 61 THAs in 53 patients were randomized to receive either a HA, FA, or uncoated Mallory-Head Porous stem during the years 1992 to 1994. Primary outcome was stem migration measured using RSA and secondary outcome was the Harris Hip Score (HHS). Evaluation took place preoperatively and postoperatively on the second day, at 6, 12, 25 and 52 weeks, and annually thereafter. At the 25-year follow-up, 12 patients (17 THAs) had died and 1 patient (1 THA) was lost to follow-up. Due to the high number of missing second-day postoperative RSA radiographs, the 1-year postoperative RSA radiograph was used as baseline for the comparative analyses.

Results — Mean follow-up was 17 years (SD 6.6). All stems showed initial rapid migration with median subsidence of 0.2 mm (–0.1 to 0.6) and median retroversion of 0.9° (–3.2 to 2) at 12 months, followed by stable migration reaching a plateau phase. No stem was revised, albeit 1 stem showed continuous subsidence up to 1.5 mm. Comparing the different coatings, we could not find a statistically significant difference in overall 25-year migration (p-values > 0.05). Median subsidence at 15-year follow-up was for HA –0.1 mm (–0.4 to 0.2), for FA 0 mm (–0.1 to 0.2), and for uncoated stems 0.2 mm (–0.1 to 0.5). Median internal rotation at 15-year follow-up was for HA not available, for FA 1.1° (–0.5 to 2.6), and for uncoated stems 0° (–0.5 to 0.4). HHS were also comparable (p-values > 0.05), with at 15-year follow-up for HA 85 points (41–99), for FA 76 points (61–90), and for uncoated stems 79 points (74–90).

Interpretation — The long-term migration pattern of cementless stems using different bioactive coatings has not previously been described. No beneficial effect, or side effect at long-term follow-up of bioactive coatings, was found. The provided migration data can be used in future research to establish thresholds for acceptable migration patterns cementless stem designs.

Long-term migration data on cementless femoral stems in total hip arthroplasty (THA) is scarce, with only a few studies reporting migration measured with radiostereometric analysis (RSA) with follow-up beyond 10 years (Sesselmann et al. 2018, Critchley et al. 2020). In a prior meta-analysis we were unable to establish a threshold for acceptable early subsidence for cementless stems, because of the lack of long-term survival and migration data (Van der Voort et al. 2015). As the number of THAs being performed is still on the rise, as well as the number of relatively young patients receiving mostly cementless THAs, the burden of future failure and subsequent revision is expected to increase (Kurtz et al. 2009). Hence longevity of implants is paramount and should be scrutinized.

Although bioactive coatings for cementless stems are widely employed, their beneficial effect remains questionable (Hailer et al. 2015, Inacio et al. 2018). Pooled data from randomized and cohort studies showed no clinical benefit of hydroxyapatite (HA)-coated implants (Gandhi et al. 2009, Goosen et al. 2009, Li et al. 2013, Chen et al. 2015) and large registry studies found no difference in risk of revision surgery (Paulsen et al. 2007, Lazarinis et al. 2011, Hailer et al. 2015). A recent registry study found an overall lower risk of revision of HA-coated stems, but this was not consistent among different implant types, suggesting a significant influence of distinct design features on longevity (Inacio et al. 2018).

Bioactive coatings were introduced in the 1980s to enhance fixation by osseointegration, with HA used as the most common coating (Geesink 1989, Furlong and Osborn 1991). However, retrieval studies have shown resorption and delamination of the HA coating from the implant, which raised concerns regarding the induction of osteolysis and, ultimately, failure of the implant (Bloebaum et al. 1994, Bauer 1995). Fluorapatite (FA) was introduced as an alternative to HA with comparable biocompatibility and osteoconductive properties (Dhert et al. 1993), but with better thermostability (Lugscheider et al. 1989). Hence, FA might adhere better to the implant during the application process using a plasma-spraying technique, thereby possibly reducing resorption and delamination of the coating (Klein et al. 1994).

HA-coated implants have shown reduced migration in comparison with their uncoated counterparts (Søballe et al. 1993, Kärrholm et al. 1994b). To our knowledge, FA has not been investigated in RSA studies, or in clinical trials.

In 1991, we initiated a trial to investigate the influence of different coatings on migration of cementless stems, the first RSA study performed at our facility. Despite teething problems, patient follow-up was continued to provide long-term migration data on cementless stems in general, and bioactive coatings specifically.

## Patients and methods

### Study design

This study was initially designed in 1991 as a multi-center, single blinded, randomized controlled trial comparing the influence of different coatings on the migration and clinical outcome of cementless THA. During the pilot phase of this study logistical problems were encountered with regard to obtaining RSA radiographs at the different participating hospitals, as this was (at that time) possible at only 1 institute (Leiden University Medical Center). Subsequently, it was decided to continue as a single-center study performed at the Leiden University Medical Center. From May 1992 to May 1994, all consecutive patients scheduled to receive a cementless primary THA for osteoarthritis, either primary or secondary to a systematic inflammatory disease and younger than 65 years of age, were approached for participation in a randomized, clinical RSA study.

Included patients were randomized to 2 intervention groups receiving either an HA- or FA-coated implant and the control group received an implant without a bioactive coating. Treatment allocation was randomized with the use of a computer-generated randomization scheme and bilateral cases were allowed. The study design was single-blind; surgeons were aware of the coating used; clinical observers were blinded to the type of coating. The study was performed in compliance with the Helsinki Declaration, approval of the institutional medical ethical board was obtained, and all patients gave written informed consent.

### Surgical technique

All THAs were implanted by experienced hip surgeons, or under their direct supervision. Surgeries were performed through a direct lateral approach in the lateral decubital position, except for 2 posterolateral approaches. For RSA measurements, 1-mm tantalum markers were inserted into the proximal femur during surgery. All patients received the same rehabilitation program starting with passive and controlled active movements on the first postoperative day and mobilization with full weight-bearing on the second postoperative day, after the first RSA radiograph was obtained.

### Implants

**Figure 1.**
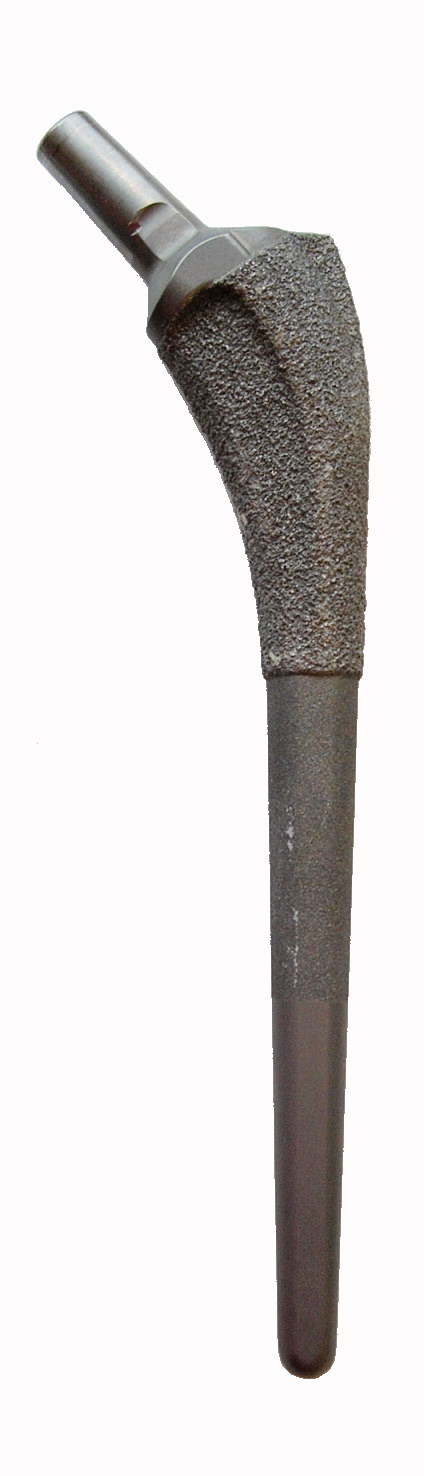
Mallory-Head Porous stem, with a porous coating on the proximal third, a grit-blasted surface on the middle third, and a smooth satin-textured surface on the distal third.

All patients received a Mallory-Head Porous stem with a dual tapered design with a round cross-sectional geometry (Biomet, Warsaw, IN, USA). The stem is characterized by an anterior and posterior flange and wide lateral fin. It is made of a titanium alloy (Ti-6A1-4V), with a porous coating on the proximal third, a grit-blasted surface on the middle third, and a smooth satin-textured surface on the distal third (Figure 1). The implants with a bioactive coating received either HA or FA plasma-sprayed onto the proximal porous coated surface. All patients received a 28 mm cobalt-chromium head and a cementless Mallory-Head finned Ringloc acetabular cup (Biomet, Warsaw, IN, USA).

### Follow-up

Patients were evaluated preoperatively and postoperatively at 6 weeks, 3 months, 6 months, 1 year, and annually thereafter. At each evaluation, RSA radiographs were obtained and the Harris Hip Score (HSS) was determined. Conventional anteroposterior and lateral radiographs were acquired preoperatively, at 6 weeks, and at 2, 5, 10, 20, 25 years postoperatively, and on indication (e.g., pain or suspected failure). On the 6-week postoperative radiographs the stem orientation (i.e., varus, neutral, or valgus) was determined. Patients unable to attend follow-up moments were contacted to check implant status and whether implant-related problems had arisen.

### RSA technique

RSA radiographs were obtained using a uniplanar setup with the patient in supine position and the calibration cage underneath the examination table. During follow-up, in 2002, the initial calibration box (Large Reference Box, Leiden, The Netherlands) was replaced by a new box (Carbon Box, Leiden, The Netherlands). Furthermore, in 2004 digital radiography was introduced. Both changes had no effect on the accuracy of the RSA measurements (Pijls et al. 2012). A marker-based analysis was carried out to calculate migration over time (Model-Based RSA software, version 3.34; RSAcore, The Netherlands), using 4 stem markers: 3 markers attached to the stem (performed by the manufacturer) and the center of the head acted as a fourth marker. Migration was expressed as translations along and rotation about 3 axes (longitudinal, transverse, and sagittal) of a right-handed orthogonal coordinate system. Since the failure mechanism of stems consists of subsidence and retroversion (Kärrholm et al. 1994a), the primary effect variables were translation along and rotation about the longitudinal axis. The accuracy of RSA measurements was determined by obtaining double examinations of 29 stems. Assuming zero migration in the brief time interval between these double examinations, the limits of the 95% prediction interval of accuracy of zero migration were determined (Table 1) (Ranstam et al. 2000). For all examinations, the mean error of rigid body fitting of the RSA markers in the femur was below 0.35 mm; the mean condition number of the RSA markers was 37 (SD 22; range, 13–111) in the femur. Bone markers were defined as unstable when they moved more than 0.5 mm with respect to the other bone markers. Unstable markers were excluded from the analyses. These values satisfy the marker stability and distribution criteria of the RSA guidelines and the ISO guideline (ISO 16087:2013) (Valstar et al. 2005).

**Table 1.** Precision of RSA measurements (upper limits of 95% zero motion confidence interval)

	Transverse (x-axis)	Longitudinal (y-axis)	Sagittal (z-axis)
Translation, mm	0.22	0.17	0.54
Rotation, °	0.80	1.13	0.31

### Statistics

Measured values of normally distributed data are reported as the mean (SD); measured values of non-normally distributed data are reported as the median (range). Estimates are reported as the mean and the 95% confidence interval (CI). Reported analyses were performed according to the per-protocol principle to reflect the genuine effect of treatment (i.e., HA vs. FA vs. uncoated). To safeguard for attrition bias, all analyses were repeated according to the intention-to-treat principle and compared with the outcomes of the per-protocol analyses. Migration and increase in HHS throughout the follow-up period were analyzed with use of a linear mixed model (LMM) with subject as a random effect. This model deals effectively with repeated measurements, missing values, and variation in duration of follow-up (DeSouza et al. 2009). Differences between the stems were assessed by estimating the main treatment effect and the stem type × time interaction, both as an overall effect over the entire follow-up period taking the repeating measurements into account. The assessment of the interaction term allows for the investigation of possible time-varying mean differences. At the 5- and 15-year follow-up point, the mean differences were assessed using ANOVA. As a sensitivity analysis, separate adjusted analyses were carried out with age, sex, BMI, and diagnosis (primary or secondary osteoarthritis) as covariates. A p-value of <0.05 was considered to be significant (SPSS version 20.0; IBM Corp, Armonk, NY, USA).

### Ethics, funding, and potential conflicts of interest

Approval from the institutional medical ethics committee was obtained and all the patients gave written informed consent. The Department of Orthopaedics of the LUMC received a single unrestricted grant from Biomet. In addition, funding from the European Information and Communication Technologies Community Seventh Framework Program (FP7/2007-2013) (grant agreement number 248693) and the Dutch Arthritis Association (project number LLP13; 08-1-300) were received in support of this study. None of these sponsors took part in the design or performance of the study, neither in the collection, management, analysis, nor in the interpretation of the data; or in preparation, review, or approval of the manuscript. None of the authors has any potential financial conflict of interest related to this manuscript.

## Results

### Patients

**Figure 2.**
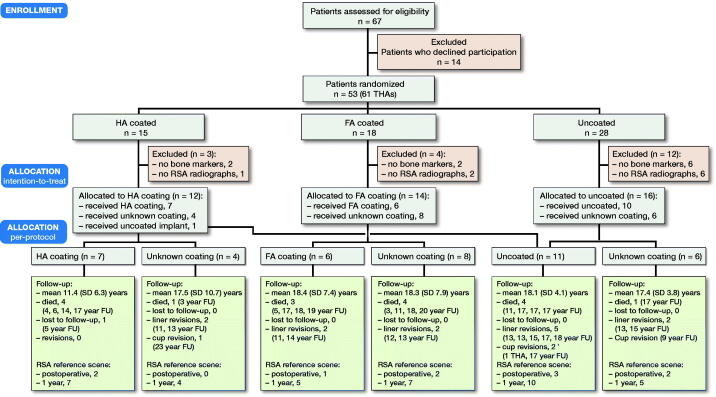
CONSORT flowchart of patient recruitment, allocation and follow-up. THA = total hip arthroplasty; HA = hydroxyapatite; FA = fluorapatite; FU = follow-up; SD = standard deviation.

From May 1992 to May 1994, 75 consecutive THAs in 67 patients were assessed for inclusion and 61 THAs in 53 patients were randomized (Figure 2). In 19 THAs (19 patients) RSA analyses could not be performed due to absence of either bone markers (n = 10) or RSA radiographs (n = 9). These 19 patients were comparable to the analyzed group with respect to sex, BMI, age at surgery, surgeon, stem orientation, and preoperative HHS (post-hoc chi-square test and unpaired Student’s t-test; p-values > 0.05). Thus, 34 patients (42 THAs) were analyzed with a mean follow-up of 17 years (SD 6.6). 7 stems were HA-coated, 6 stems were FA-coated, and 11 stems were uncoated (Table 2). 1 patient was randomized for an HA-coated stem, but instead received an uncoated stem. In 18 stems the coating was unknown due to missing implant stickers in 9 cases, missing coating details on the implant sticker in 4 cases, and missing medical (paper) records in 5 cases. 12 patients (17 THAs) died during follow-up and 1 patient (1 THA) emigrated and was subsequently lost after 5 years of follow-up.

**Table 2.** Group characteristics at baseline. Values are count unless otherwise specified

	Hydroxy- apatite	Fluor- apatite	Uncoated	Unknown
Characteristic	(n = 7)	(n = 6)	(n = 11)	(n = 18)
Sex				
Male	3	1	5	7
Female	4	5	6	11
BMI ^a^	23 (5.6)	28 (2.7)	24 (4.5)	25 (3.3)
Age at surgery ^a^	58 (15)	57 (13)	50 (14)	53 (9)
Diagnosis				
Osteoarthritis	5	2	4	6
Rheumatoid arthritis	1	2	4	9
Hip dysplasia	0	1	1	1
Ankylosing spondylitis	1	1	1	0
Osteonecrosis	0	0	0	2
Perthes disease	0	0	1	0
Side				
Left	4	5	5	6
Right	3	1	6	12
Surgeon				
Consultant	7	6	10	14
Resident	0	0	1	4
Stem orientation				
Varus	0	1	0	1
Neutral (< 3°)	7	5	10	16
Valgus	0	0	1	1
Preoperative HHS ^a^				
min 0–max 100 points	33 (18)	34 (4.5)	32 (14)	37 (15)

**^a^**Values are mean (SD).

### Migration

**Figure 3.**
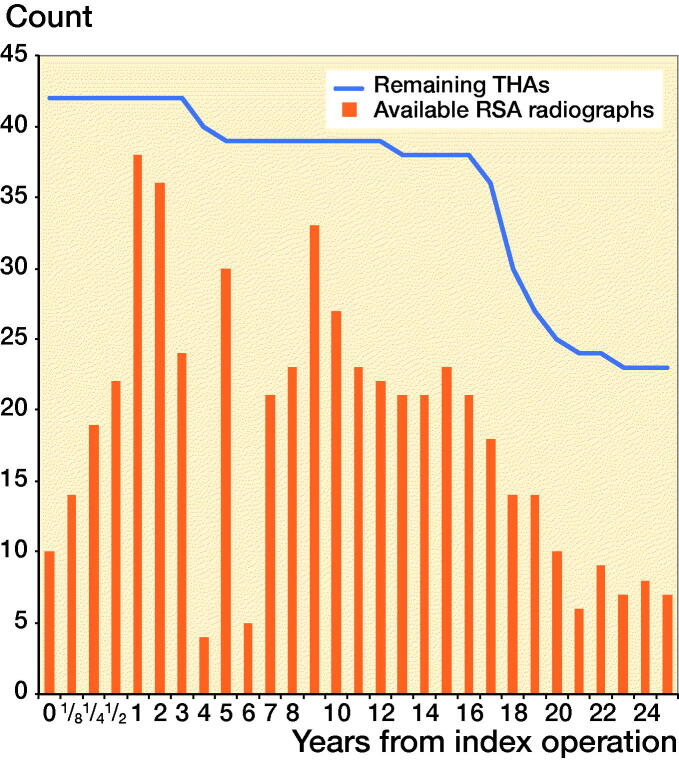
Bar graph showing number of RSA radiographs available for analysis per follow-up point. Line graph showing number of THAs in follow-up (i.e., total minus deceased and lost to follow-up).

**Figure 4.**
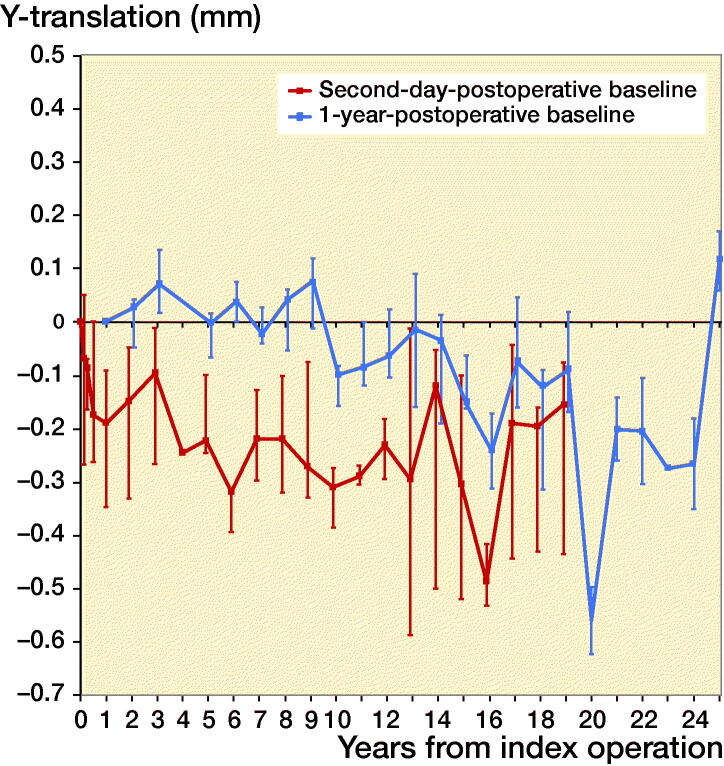
Median Y-translation (i.e., translation along the longitudinal axis) with interquartile ranges of the complete cohort during the 25 years of follow-up, using both the second-day (n = 10) and the 1-year (n = 38) postoperative RSA radiograph as a baseline.

**Figure 5.**
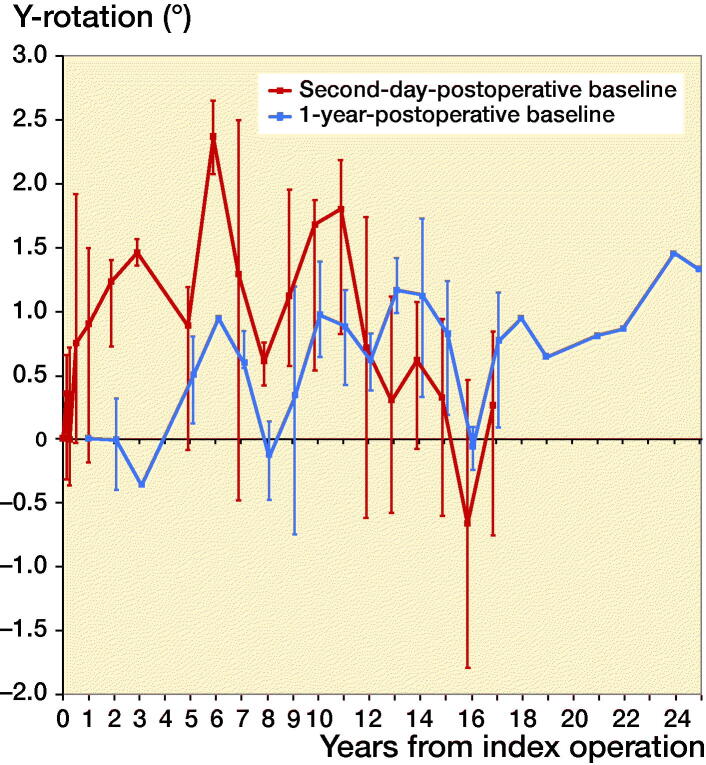
Median Y-rotation (i.e.,internal rotation about the longitudinal axis) with interquartile ranges of the complete cohort during the 25 years of follow-up, using the both the second-day (n = 6) and the 1-year (n = 17) postoperative RSA radiograph as a baseline.

Second-day postoperative RSA radiographs were available in only 10 out of 42 stems, whereas RSA radiographs at 1-year follow-up were available in 38 out of 42 stems (Figure 3). As the number of available second-day postoperative RSA radiographs was not sufficient to make meaningful comparative analyses between different coatings, the 10 stems available for direct postoperative migration measurement were analyzed as a single cohort, independent of coating, showing relatively rapid subsidence during the first postoperative year with a median subsidence of 0.2 mm (–0.1 to 0.6) at 12 months, followed by stable subsidence during the remaining follow-up period (Figure 4). During the period of initial subsidence there were also relatively large rotations of these stems in the horizontal plane, which stabilized after 1 year (Figure 5).

**Figure 6.**
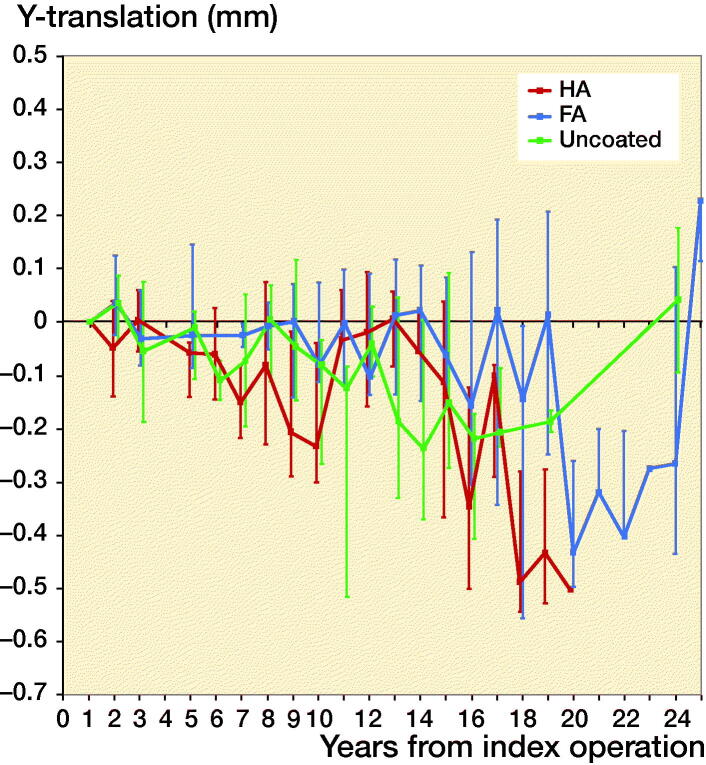
Median Y-translation (i.e., translation along the longitudinal axis) with interquartile ranges during the 25 years of follow-up of the HA, FA, and uncoated stems, using intention-to-treat analysis (i.e., all included stems as per randomization group) and the 1-year postoperative RSA radiograph as a baseline (i.e., unknown initial migration).

**Figure 7.**
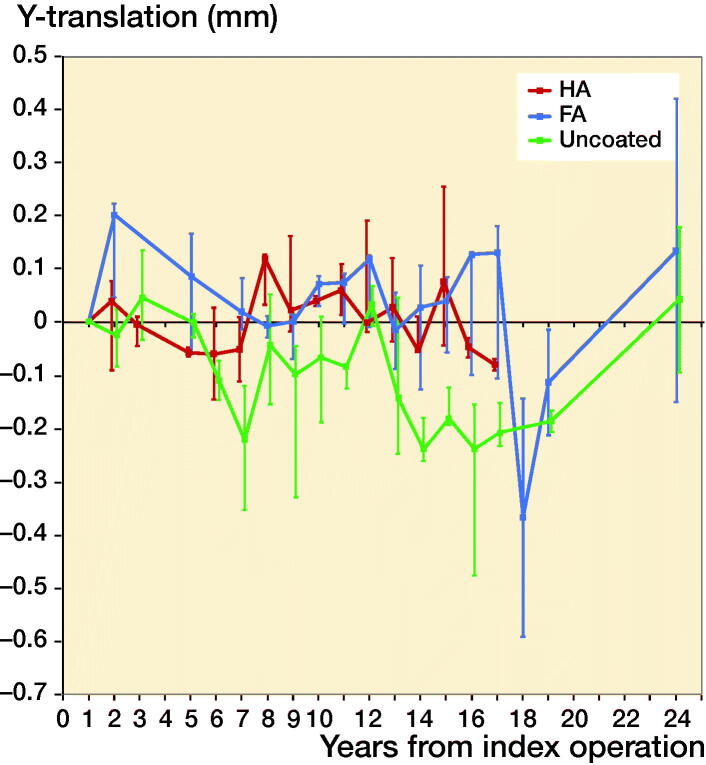
Median Y-translation (i.e., translation along the longitudinal axis) with interquartile ranges during the 25 years of follow-up of the HA, FA, and uncoated stems, using per protocol analysis (i.e., only stems with verified coating) and the 1-year postoperative RSA radiograph as a baseline (i.e., unknown initial migration).

The 38 stems available for migration measurement using the 1-year postoperative RSA radiograph as baseline were analyzed both as a single cohort, and in a comparative manner using different coatings. Migration of the overall cohort showed rather stable subsidence and rotation until 14 years’ follow-up, after which the migration patterns began to show large variability (Figures 4 and 5). Also, from this time period onward the number of patients attending the RSA outpatient clinic began to decline (Figure 3). For the comparative migration analyses (i.e., HA vs. FA vs. uncoated stems), both intention-to-treat (ITT) and per-protocol (PP) analyses were performed. The ITT analyses reflect the best-case scenario, using allocation as per randomization group of 42 THAs (including both 24 THAs with verified and 18 THAs with unknown coating) (Figure 6), whereas per-protocol (PP) analyses reflect the genuine effect of the different coatings, including only the 24 THAs with verified coating and excluding the 18 THAs with unknown coating (Figure 7). Post-hoc verification revealed adequate randomization in both ITT and PP analyses as the 3 different coatings coating groups were comparable with respect to sex, BMI, age at surgery, surgeon, stem orientation, and preoperative HHS (post-hoc chi-square test and 1-way ANOVA; p-values > 0.05). Overall 25-year migration did not reveal a significant difference among the 3 different coatings in both the ITT and PP analyses (LMM; p-values > 0.05; Table 3; Tables 4 and 5 in Supplementary data). Furthermore, we could not find a significant difference in time to stabilization and subsequent migration, that is, no evidence of interaction (coating type × time interaction; LMM; p-values > 0.05; Tables 4 and 5 in Supplementary data). Migration of the stems was comparable among the 3 coatings at the prespecified time points of 5 years and 15 years postoperatively (1-way ANOVA; p-values > 0.05; Table 3). The results of the adjusted analyses were comparable to the results from the unadjusted analyses and age, sex, diagnosis, and BMI did not significantly influence migration.

**Table 3.** Stem migration during 25 years of follow-up: per-protocol analysis (i.e., only stems with verified coating), using the 1-year postoperative RSA radiograph as a baseline (i.e., unknown initial migration). Values are count, median (range)

FU year	Hydroxyapatite		Fluorapatite		Uncoated		p-value
Longitudinal translation, mm ^a^							
2	7	0.04 (–0.19 to 0.32)	5	0.20 (–0.02 to 0.27)	8	–0.03 (–0.16 to 0.12)	0.1 ^b^
5	2	–0.06 (–0.07 to –0.04)	5	0.09 (–0.16 to 0.44)	6	0.00 (–0.12 to 0.12)	0.6 ^c^
10	2	0.04 (0.02 to 0.06)	4	0.07 (–0.08 to 0.12)	7	–0.07 (–0.41 to 0.37)	
15	3	0.07 (–0.16 to 0.44)	3	0.04 (–0.15 to 0.13)	5	–0.18 (–0.52 to 0.12)	0.5 ^c^
20	0	–	1	–	1	–	
25	0	–	1	–	1	–	
Internal rotation, degrees							
2	3	–0.21 (–1.35 to 0.24)	2	–1.02 (–1.23 to –0.81)	3	–0.50 (–0.76 to 0.25)	0.5 ^b^
5	1	–	2	–0.53 (–0.74 to –0.32)	2	–0.10 (–0.48 to 0.28)	–
10	1	–	2	–0.14 (–0.22 to –0.05)	2	–0.33 (–0.52 to –0.13)	
15	0	–	2	1.08 (–0.46 to 2.62)	2	–0.02 (–0.45 to 0.42)	–
20	0	–	0	–	0	–	
25	0	–	0	–	0	–	

**^a^**Negative values correspond to subsidence (i.e., distal migration).

**^b^**Main effect, per protocol

**^c^**Prespecified time point, per protocol

**Table 6.** Harris Hip Score (min 0 – max 100 points) during 25 years of follow-up: per-protocol analysis (i.e., only stems with verified coating). Values are count, median (range)

	Hydroxyapatite		Fluorapatite		Uncoated		p-value
Preoperative	5	35 (8–57)	3	34 (30–39)	9	37 (8–54)	0.6 ^a^
Year 2	2	81(65–96)	2	90 (89–91)	4	95 (85–100)	
Year 5	2	100 (99–100)	3	76 (69–84)	5	85 (66–100)	
Year 10	4	86 (72–94)	5	73 (62–90)	7	86 (61–98)	
Year 15	4	85 (41–99)	5	76 (61–90)	6	79 (74–90)	0.9 ^b^
Year 20	0	–	2	63 (57–69)	2	83 (82–83)	
Year 25	0	–	1	–	1	–	

**^a^**Main effect, per protocol

**^b^**Prespecified time point, per protocol

**Figure 8.**
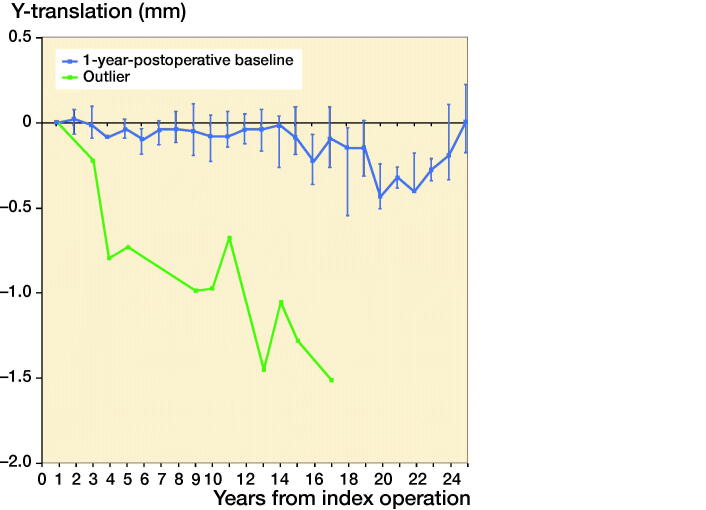
Median Y-translation (i.e., translation along the longitudinal axis) with interquartile ranges of the complete cohort during the 25 years of follow-up with the influential outlier excluded and shown separately.

Subsidence in 1 stem did not stabilize (Figure 8). This stem was randomized for no coating, but as the implant sticker was missing this could not be verified. At the last available radiograph after 17 years’ follow-up there was evidence of subsidence, and around the tip of the stem radiolucencies and pedestal formation were noticeable. However, the HHS remained higher than 90 points during follow-up.

### Clinical outcome

We could not find a significant difference in HHS among the groups during follow-up in both the PP and ITT analyses (LMM; p-values > 0.05; Table 6; Tables 7 and 8 in Supplementary data). Between-group differences did not change significantly over time (coating type × time interaction; LMM; p-values > 0.05; Tables 7 and 8 in Supplementary data). Adjusted analyses for age, sex, diagnosis, BMI, and postoperative HHS gave similar results. Furthermore, comparing the HHS at the 15-year follow-up point did not yield any statistically significant differences (1-way ANOVA; p-values > 0.05; Table 6).

### Survival

None of the stems were revised during follow-up. There were 13 liner revisions in 13 THAs due to wear. In 1 THA (uncoated) with a liner revision 13 years after follow-up, the cup was revised due to aseptic loosening 20 years after follow-up and subsequently re-revised a couple weeks later due to malpositioning. In addition, 2 more cups in 2 THAs (coating unknown) were revised due to aseptic loosening. During follow-up, 12 patients (17 THAs) died due to causes unrelated to the THA. One patient (1 THA) was lost to follow-up due to emigration, the fate of this THA could not be determined.

## Discussion

We found stable migration and thus fixation of the cementless Mallory-Head Porous stem over a period of 25 years. After initial migration, all but 1 of 42 stems stabilized after 1-year follow-up and there were no stem revisions. 4 cups were revised due to aseptic loosening and 13 liners were revised due to wear. Comparative analyses did not yield a difference in migration and clinical scores among HA, FA, and uncoated cementless stems. Furthermore, there was no difference in time to stabilization between coated and uncoated stems, thus excluding a delamination problem occurring later in follow-up.

To our knowledge, this is the first long-term RSA study with over 20 years’ follow-up, and the first RSA study comparing migration of HA, FA, and uncoated stems. Only a few RSA studies describe migration beyond 10-year follow-up. Sesselman et al. (2018) reported migration of 26 cementless Cerafit stems with a follow-up of 10 years. They found a median subsidence of 0.01 mm at 2-year and 0.09 mm at 10-year follow-up, with most of the subsidence occurring during the first 6 postoperative weeks. Critchley et al. (2020) reported migration of 30 cementless Corail stems with a follow-up of 14 years. They found a mean subsidence of 0.62 mm at 2-year and 0.7 mm at 14-year follow-up, with initial rapid subsidence over 6 weeks and subsequent stabilization. In our study subsidence was 0.15 mm at 2-year, 0.3 mm at 10-year, and 0.1 mm at 14-year follow-up, with most of the subsidence occurring during the first 2 postoperative years. These studies, using different cementless stem designs, all show initial migration of different magnitude with subsequent stabilization, emphasizing the influence of design features on migration, and thus fixation.

Søballe et al. (1993) and Kärrholm et al. (1994b) performed RSA studies comparing the migration of HA-coated stems with stems without bioactive coating. Søballe et al. (1993) found more migration (MTPM) of uncoated titanium stems compared with HA-coated stems, although subsidence was comparable after 1-year follow-up with a mean of 0.09 mm for the HA-coated stems. Kärrholm et al. (1994b) compared migration of HA-coated stems with cemented and cementless stems over a 2-year period. They found a median of 0.05 mm proximal migration of HA-coated stems compared with 0.12 mm subsidence of cemented and 0.1 mm of cementless stems. As these differences were statistically significant both authors concluded that HA seems to enhance early fixation. In our study we could not find a benefit of HA on long-term fixation. There might be a benefit of HA during the first postoperative year. However, due to insufficient data we were unable to find such a difference. Our study shows that once a stem has stabilized there is no difference in migration among the different coatings.

Several reviews have been published comparing HA-coated implants with uncoated implants. Gandhi et al. (2009) reported no difference in aseptic loosening, or in HHS. Goosen et al. (2009) reported no difference in HHS, endosteal bone ingrowth, and radiolucent lines. Li et al. (2013) could not find a difference in HHS, or radioactive lines, and Chen et al. (2015) reported no benefit of HA in terms of survivorship, but HA-coated implants showed better postoperative HHS and less femoral osteolysis during follow-up.

A recent registry study found an overall lower risk of stem revision for any reason for HA-coated stems compared with a non-HA coated stem; however, the rate for stem revision for HA-coated Mallory-Head stems was higher compared with the non-HA-coated counterpart (0.11% vs. 0.02%) (Inacio et al. 2018). This study suggests that longevity of implants might be related more to specific implant design than to type of coating.

Except for 1 study performed at our institution, there are no RSA studies evaluating the migration of the Mallory-Head Porous stem. Van der Voort et al. (in submission) showed median subsidence of 0.2 mm (range 0.4–4.8) at 5 years’ follow-up of uncoated stems. In the current study we found the same median subsidence at 5 years’ follow-up but the range in this study was considerably smaller, with the largest subsidence being only 0.4 mm. Insufficient data on initial migration during the first postoperative year in this study might explain this difference.

The cementless Mallory-Head Porous stem has an excellent 10-year survival record with 48 stem revisions of 5,932 primary THAs in the Dutch Arthroplasty Register (LROI 2019) and 27 stem revisions of 3,303 primary THAs in the Australian Orthopaedic Association National Joint Replacement Registry (AOANJRR 2019).

All stems in our study showed stable subsidence, except for 1. This stem, with an untraceable coating, was inserted because of severe osteoarthritis at the age of 61 years. There was no preoperative template available but the postoperative radiograph showed a varus position of the stem with insufficient contact with the lateral cortex at the metaphysis, suggesting undersizing. Albeit that initial subsidence was unknown, the stem showed progressive subsidence from the 1-year follow-up onwards. On the 17-year follow-up radiograph there was obvious subsidence visible, next to radiolucencies and pedestal formation. Remarkably, this patient never scored less than 90 points on the HHS scale and at the 17-year follow-up moment the patient was asymptomatic, although, aged 78 years, she walked only about 200 meters.

Overall, there was rather stable migration up to 14 years reaching a plateau phase, but thereafter migration patterns began to show greater variability, which especially holds for subsidence. From then onwards, patient attendance for regular follow-up also decreased dramatically, resulting in only 5 stems being available for analysis at 20 years’ follow-up. This low number of available, analog RSA radiographs in combination with relative low precision compared with the modern RSA technique are the most probable reasons for the great variability in stem migration patterns (Valstar et al. 2000). Furthermore, as most stems available for analyses beyond 20 years were FA coated, it could be reasoned that in this selected group of patients either overall subsidence increased or the FA coating broke down after more than a decade, leading to increased subsidence. However, the latter cannot be substantiated as no data on the non-coated control group was available. Additionally, the increasing retroversion, together with to increasing subsidence, might be related to a more sedentary lifestyle of these slightly older patients. For that matter, standing up from a chair creates a retroversion force at the neck of the femoral stem.

This was the first RSA study performed at our institution, therefore it gave some insights into initial study set-up logistics. There was experience neither with inserting tantalum markers in periprosthetic bone, nor with the validity of the instrument used at that time to insert markers, which turned out to skip 1 out of 4 markers. This was noticed only after RSA radiographs were evaluated in too late a postoperative period. For that reason a novel tantalum marker inserter was developed. Additionally, there was a lack of the expertise needed for optimal logistics concerning RSA radiographs. Initially, a single researcher (RN) took care of study logistics and RSA radiograph analyses without secretarial assistance. Hence, patients missing a follow-up moment were noticed only weeks later. These technical and logistical shortcomings resulted in the exclusion of 19 THAs. Furthermore, stem allocation to the randomization groups was not adequately documented and implant stickers of the manufacturer were missing or lacking essential information. The latter might have been related to the distinctive manufacturing process for stems in the current study; the attachment of 3 RSA markers and applying 3 different coatings might have interfered with regular application of implant stickers.

This study has several limitations. First, there were too few migration measurements available during the first postoperative year to make meaningful analyses using the second-day postoperative radiograph as baseline, which is the conventional manner to calculate migration over time. However, as stems susceptible to failure will typically show progressive migration, using the 1-year postoperative RSA radiograph will also detect stems prone to failure. Second, the given coating could not be verified in 18 stems due to due to missing or insufficient implant stickers. To overcome this problem of unknown coatings, both per-protocol and intention-to-treat analyses were performed. The latter reflects the base case scenario, assuming all stems received the coating as per randomization. This is a plausible assumption as only 1 of 24 known coatings received a different coating as per randomization. Third, the Mallory-Head Porous stem is nowadays seldom used, limiting the clinical applicability of this study.

In conclusion, this study could not establish a beneficial effect at long-term follow-up of bioactive coatings on migration, and thus fixation, in this type of stem. Neither could delamination of the less thermostable HA coating be proven. This study provides value migration data that can be used to establish an acceptable migration pattern of cementless stems with which new stem designs can be compared.

## Supplementary Material

Supplemental Material

## Supplementary data

Tables 4–5 and 7–8 are available as supplementary data in the online version of this article, http://dx.doi.org/10.1080/17453674.2020.1840021
