# Supplementary material for: Long-term migration of a cementless stem with different bioactive coatings. Data from a “prime” RSA study: lessons learned
Source: Acta Orthop. 2020 Nov 4;91(6):660–8. doi: 10.1080/17453674.2020.1840021 (PMC8023916; doi:10.1080/17453674.2020.1840021)
Supplement: Supplemental Material [file IORT_A_1840021_SM0489.pdf]

Supplementary data

Table 4. Stem migration during 25 years of follow-up. Per-protocol analysis (i.e., only stems with verified coating), using the 1-year postoperative RSA radiograph as a baseline (i.e., unknown initial migration). Values are count, mean (SD), median (range)

Migration FU year	Hydroxyapatite			Fluorapatite			Uncoated			p-value	
Translation, mm:											
x-axis (medial–lateral)											
2	7	-0.07 (0.29)	-0.16 (-0.49 to 0.45)	5	-0.09 (0.17)	0.00 (-0.37 to 0.05)	8	0.01 (0.22)	-0.08 (-0.16 to 0.50)	0.5 [a]	1.0 [b]
5	2	-0.39 (0.40)	-0.39 (-0.67 to -0.10)	5	-0.17 (0.35)	-0.06 (-0.78 to 0.13)	6	0.01 (0.12)	0.02 (-0.13 to 0.15)		0.2 [c]
10	2	-0.25 (0.17)	-0.25 (-0.37 to -0.13)	4	0.00 (0.11)	-0.03 (-0.10 to 0.17)	7	0.09 (0.22)	0.07 (-0.19 to 0.49)		
15	3	-0.20 (0.09)	-0.22 (-0.28 to -0.09)	3	0.02 (0.10)	-0.01 (-0.06 to 0.13)	5	-0.01 (0.13)	-0.02 (-0.17 to 0.13)		0.5 [c]
20	0	–	–	1	–	–	1	–	–		
25	0	–	–	1	–	–	1	–	–		
y-axis (cranial–caudal) [d]											
2	7	0.02 (0.17)	0.04 (-0.19 to 0.32)	5	0.14 (0.13)	0.20 (-0.02 to 0.27)	8	-0.03 (0.09)	-0.03 (-0.16 to 0.12)	0.1 [a]	1.0 [b]
5	2	-0.06 (0.02)	-0.06 (-0.07 to -0.04)	5	0.09 (0.23)	0.09 (-0.16 to 0.44)	6	0.00 (0.08)	0.00 (-0.12 to 0.12)		0.6 [c]
10	2	0.04 (0.03)	0.04 (0.02 to 0.06)	4	0.05 (0.09)	0.07 (-0.08 to 0.12)	7	-0.07 (0.25)	-0.07 (-0.41 to 0.37)		
15	3	0.12 (0.30)	0.07 (-0.16 to 0.44)	3	0.00 (0.14)	0.04 (-0.15 to 0.13)	5	-0.18 (0.23)	-0.18 (-0.52 to 0.12)		0.5 [c]
20	0	–	–	1	–	–	1	–	–		
25	0	–	–	1	–	–	1	–	–		
z-axis (anterior–posterior)											
2	7	0.20 (0.76)	-0.09 (-0.61 to 1.56)	5	0.51 (0.45)	0.42 (0.06 to 1.21)	8	0.04 (0.15)	0.09 (-0.29 to 0.15)	0.08 [a]	0.5 [c]
5	2	-0.12 (0.19)	-0.12 (-0.26 to 0.01)	5	0.71 (0.63)	0.66 (-0.04 to 1.63)	6	0.29 (0.36)	0.28 (-0.19 to 0.89)		0.05 [c]
10	2	0.10 (0.21)	0.10 (-0.05 to 0.25)	4	0.14 (0.26)	0.13 (-0.16 to 0.46)	7	0.07 (0.47)	0.18 (-0.74 to 0.59)		
15	3	0.34 (0.45)	0.19 (-0.01 to 0.85)	3	0.49 (0.17)	0.57 (0.29 to 0.60)	5	-0.17 (0.38)	-0.06 (-0.81 to 0.16)		0.3 [c]
20	0	–	–	1	–	–	1	–	–		
25	0	–	–	1	–	–	1	–	–		
Rotation, degrees											
x-axis (transverse)											
2	3	0.13 (0.62)	0.00 (-0.42 to 0.80)	2	-0.04 (0.28)	-0.04 (-0.24 to 0.16)	3	0.18 (0.36)	0.15 (-0.17 to 0.54)	0.5 [a]	0.9 [b]
5	1	–	–	2	-0.32 (0.28)	-0.32 (-0.52 to -0.12)	2	0.20 (0.03)	0.20 (0.18 to 0.21)		–
10	1	–	–	2	0.12 (0.28)	0.12 (-0.07 to 0.32)	2	0.62 (0.89)	0.62 (-0.01 to 1.25)		
15	0	–	–	2	-0.57 (0.42)	-0.57 (-0.86 to -0.27)	2	0.27 (0.43)	0.27 (-0.03 to 0.58)		–
20	0	–	–	0	–	–	0	–	–		
25	0	–	–	0	–	–	0	–	–		
y-axis (longitudinal) [e]											
2	3	-0.44 (0.82)	-0.21 (-1.35 to 0.24)	2	-1.02 (0.30)	-1.02 (-1.23 to -0.81)	3	-0.34 (0.53)	-0.50 (-0.76 to 0.25)	0.5 [a]	0.2 [b]
5	1	–	–	2	-0.53 (0.30)	-0.53 (-0.74 to -0.32)	2	-0.10 (0.53)	-0.10 (-0.48 to 0.28)		–
10	1	–	–	2	-0.14 (0.12)	-0.14 (-0.22 to -0.05)	2	-0.33 (0.28)	-0.33 (-0.52 to -0.13)		
15	0	–	–	2	1.08 (2.18)	1.08 (-0.46 to 2.62)	2	-0.02 (0.62)	-0.02 (-0.45 to 0.42)		–
20	0	–	–	0	–	–	0	-	-		
25	0	–	-	0	–	–	0	-	-		
z-axis (sagittal)											
2	3	-0.07 (0.46)	0.15 (-0.60 to 0.23)	2	0.18 (0.11)	0.18 (0.11 to 0.26)	3	0.09 (0.17)	0.18 (-0.11 to 0.19)	0.3 [a]	1.0 [b]
5	1	–	–	2	0.12 (0.06)	0.12 (0.07 to 0.16)	2	0.01 (0.19)	0.01 (-0.13 to 0.15)		–
10	1	–	–	2	0.09 (0.04)	0.09 (0.06 to 0.12)	2	-0.06 (0.09)	-0.06 (-0.12 to 0.00)		
15	0	–	–	2	0.05 (0.34)	0.05 (-0.19 to 0.29)	2	0.03 (0.01)	0.03 (0.02 to 0.04)		–
20	0	–	–	0	–	–	0	–	–		
25	0	–	–	0	–	–	0	–	–		

[a] Main effect, per protocol
[b] Group × time interaction, per protocol
[c] Prespecified time point, per protocol
[d] Negative values correspond to subsidence.
[e] Positive values correspond to internal rotation.

 Acta Orthopaedica 2020; 91 (DOI: 10.1080/17453674.2020.1840021)

Table 5. Stem migration during 25 years of follow-up. Intention-to-treat analysis (i.e., all included stems as per randomization group), using the 1-year postoperative RSA radiograph as a baseline (i.e., unknown initial migration). Values are count, mean (SD), median (range)

Migration FU year	Hydroxyapatite			Fluorapatite			Uncoated			p-value	
Translation, mm:											
x-axis (medial–lateral)											
2	12	-0.01 (0.28)	-0.03 (-0.49 to 0.50)	12	-0.05 (0.13)	-0.01 (-0.37 to 0.15)	11	0.04 (0.17)	0.06 (-0.16 to 0.30)	0.8 [a]	1 [b]
5	6	-0.15 (0.29)	-0.11 (-0.67 to 0.13)	10	-0.08 (0.27)	-0.05 (-0.78 to 0.17)	10	0.07 (0.34)	0.09 (-0.63 to 0.70)		0.3 [c]
10	6	-0.01 (0.22)	0.01 (-0.37 to 0.20)	9	-0.09 (0.25)	-0.03 (-0.62 to 0.17)	10	0.15 (0.64)	0.11 (-1.08 to 1.48)		
15	6	-0.17 (0.09)	-0.17 (-0.28 to -0.05)	7	-0.09 (0.27)	-0.03 (-0.70 to 0.13)	8	-0.17 (0.49)	-0.05 (-1.34 to 0.13)		0.9 [c]
20	2	-0.11 (0.00)	0.00 (-0.11 to -0.11)	4	-0.32 (0.38)	-0.32 (-0.72 to 0.10)	1	–	–		
25	1	–	–	3	0.01 (0.10)	0.02 (-0.10 to 0.10)	1	–	–		
y-axis (cranial–caudal) [d]											
2	12	-0.03 (0.15)	-0.05 (-0.19 to 0.32)	12	0.06 (0.12)	0.04 (-0.15 to 0.27)	11	0.02 (0.11)	0.04 (-0.16 to 0.21)	0.5 [a]	0.9 [b]
5	6	-0.09 (0.08)	-0.06 (-0.23 to -0.02)	10	0.06 (0.23)	-0.02 (-0.17 to 0.44)	10	-0.06 (0.28)	-0.01 (-0.73 to 0.36)		0.4 [c]
10	6	-0.19 (0.19)	-0.23 (-0.41 to 0.06)	9	-0.06 (0.19)	-0.08 (-0.50 to 0.12)	10	-0.21 (0.40)	-0.08 (-0.98 to 0.37)		
15	6	-0.12 (0.37)	-0.11 (-0.59 to 0.44)	7	-0.03 (0.21)	-0.06 (-0.32 to 0.33)	8	-0.23 (0.48)	-0.15 (-1.28 to 0.24)		0.6 [c]
20	2	-0.50 (0.02)	0.00 (-0.52 to -0.49)	4	-0.32 (0.41)	-0.43 (-0.69 to 0.26)	1	–	–		
25	1	–	–	3	0.15 (0.13)	0.23 (0.00 to 0.23)	1	–	–		
z-axis (anterior–posterior)											
2	12	0.25 (0.61)	0.13 (-0.61 to 1.56)	12	0.19 (0.58)	0.18 (-1.17 to 1.21)	11	0.11 (0.22)	0.13 (-0.29 to 0.60)	0.3 [a]	0.9 [b]
5	6	0.24 (0.52)	0.12 (-0.26 to 1.18)	10	0.22 (0.79)	0.18 (-1.34 to 1.63)	10	0.02 (0.43)	0.03 (-0.67 to 0.89)		0.7 [c]
10	6	0.10 (0.23)	0.01 (-0.13 to 0.50)	9	0.03 (0.32)	0.08 (-0.55 to 0.46)	10	-0.07 (0.49)	-0.07 (-0.74 to 0.60)		
15	6	0.38 (0.46)	0.18 (-0.01 to 1.05)	7	0.15 (0.52)	0.29 (-0.78 to 0.60)	8	-0.23 (0.46)	-0.10 (-0.93 to 0.36)		0.8 [c]
20	2	-0.13 (0.31)	0.00 (-0.35 to 0.09)	4	0.17 (0.98)	-0.10 (-0.69 to 1.58)	1	–	–		
25	1	–	–	3	0.32 (0.38)	0.54 (-0.12 to 0.54)	1	–	-–		
Rotation, degrees											
x-axis (transverse)											
2	5	-0.31 (0.86)	-0.36 (-1.57 to 0.80)	5	-0.57 (1.04)	-0.24 (-2.41 to 0.16)	5	-0.10 (0.46)	-0.17 (-0.59 to 0.54)	0.8 [a]	0.5 [b]
5	3	-0.63 (1.64)	0.07 (-2.49 to 0.55)	4	-0.08 (0.36)	-0.07 (-0.52 to 0.34)	5	-0.20 (0.68)	0.18 (-1.30 to 0.33)		0.7 [c]
10	3	0.45 (0.27)	0.35 (0.23 to 0.75)	5	-0.38 (0.96)	-0.07 (-2.08 to 0.32)	4	0.44 (0.69)	0.37 (-0.23 to 1.25)		
15	2	-0.81 (1.69)	-0.81 (-2.01 to 0.39)	4	-0.05 (0.70)	-0.07 (-0.86 to 0.81)	4	-0.08 (0.70)	0.08 (-1.07 to 0.58)		0.6 [c]
20	1	–	–	0	–	–	0	–	–		
25	1	–	–	1	–	–	0	–	–		
y-axis (longitudinal) [e]											
2	5	0.08 (0.93)	0.24 (-1.35 to 1.11)	5	-0.37 (0.77)	-0.37 (-1.23 to 0.82)	5	-0.18 (0.54)	-0.41 (-0.76 to 0.50)	0.4 [a]	0.6 [b]
5	3	0.59 (1.13)	0.57 (-0.53 to 1.74)	4	0.14 (0.85)	0.07 (-0.74 to 1.18)	5	0.26 (0.44)	0.32 (-0.48 to 0.68)		0.8 [c]
10	3	-0.02 (0.89)	-0.47 (-0.59 to 1.01)	5	0.03 (0.73)	-0.05 (-0.94 to 1.05)	4	0.67 (1.31)	0.39 (-0.52 to 2.42)		
15	2	1.17 (0.24)	1.17 (1.00 to 1.34)	4	0.52 (1.59)	0.19 (-0.92 to 2.62)	4	0.51 (0.86)	0.42 (-0.45 to 1.65)		0.8 [c]
20	1	–	–	0	–	–	0	–	–		
25	1	–	–	1	–	–	0	–	–		
z-axis (sagittal)											
2	5	-0.24 (0.58)	0.09 (-1.09 to 0.23)	5	-0.03 (0.38)	0.11 (-0.69 to 0.26)	5	0.00 (0.18)	-0.08 (-0.20 to 0.19)	0.7 [a]	0.5 [b]
5	3	-0.33 (0.88)	0.14 (-1.34 to 0.23)	4	-0.02 (0.19)	0.02 (-0.28 to 0.16)	5	-0.07 (0.25)	0.03 (-0.48 to 0.15)		0.7 [c]
10	3	0.16 (0.05)	0.16 (0.11 to 0.20)	5	-0.18 (0.33)	-0.01 (-0.56 to 0.12)	4	-0.03 (0.07)	-0.02 (-0.12 to 0.05)		
15	2	-0.57 (1.07)	-0.57 (-1.32 to 0.18)	4	0.08 (0.23)	0.10 (-0.19 to 0.29)	4	-0.24 (0.37)	-0.11 (-0.76 to 0.04)		0.4 [c]
20	1	–	–	0	–	–	0	–	–		
25	1	–	–	1	–	–	0	–	–		

[a] Main effect, intention to treat
[b] Group × time interaction, intention to treat
[c] Prespecified time point, intention to treat
[d] Negative values correspond to subsidence.
[e] Positive values correspond to internal rotation.

Table 7. Harris Hip Score (min 0 – max 100 points) during 25 years of follow-up, per-protocol analysis (i.e., only stems with verified coating). Values are count, mean (SD), median (range)

FU year	Hydroxyapatite			Fluorapatite			Uncoated			p-value	
Preop.	5	33 (17)	35 (8–57)	3	34 (5)	34 (30–39)	9	32 (14)	37 (8–54)	0.6 [a]	0.3 [b]
2	2	81 (22)	81 (65–96)	2	90 (1)	90 (89–91)	4	94 (6)	95 (85–100)		
5	2	99 (1)	99 (99–100)	3	76 (8)	76 (69–84)	5	84 (13)	85 (66–100)		
10	4	85 (10)	86 (72–94)	5	75 (13)	73 (62–90)	7	81 (14)	86 (61–98)		
15	4	77 (25)	85 (41–99)	5	76 (13)	76 (61–90)	6	80 (6)	79 (74–90)		0.9 [c]
20	0	–	–	2	63 (8)	63 (57–69)	2	83 (1)	83 (82–83)		
25	0	–	–	1	–	–	1	–	–		

[a] Main effect, per protocol
[b] Group × time interaction, per protocol
[c] Prespecified time point, per protocol

Table 8. Harris Hip Score (min 0 – max 100 points) during 25 years of follow-up, intention-to-treat analysis (i.e., all included stems as per randomization group). Values are count, mean (SD), median (range)

FU year	Hydroxyapatite			Fluorapatite			Uncoated			p-value	
Preop.	7	32 (15)	31 (8–57)	4	31 (8)	32 (21–39)	11	36 (15)	37 (8–61)	1 [a]	0.3 [b]
2	4	84 (15)	87 (65–96)	4	81 (12)	84 (64–91)	6	92 (10)	96 (74–100)		
5	4	97 (5)	99 (90–100)	9	84 (10)	84 (69–96)	8	83 (14)	83 (66–100)		
10	8	90 (9)	93 (72–100)	10	83(15)	88 (62–100)	11	79 (17)	86 (54–98)		
15	7	77 (24)	89 (41–99)	8	82 (13)	89 (61–95)	10	79 (9)	79 (60–91)		0.8 [c]
20	1	–	–	5	71 (11)	69 (57–87)	3	84 (3)	83 (82–87)		
25	1	–	–	4	86 (6)	87 (79–93)	2	78 (2)	78 (76–79)		

[a] Main effect, intention to treat
[b] Group × time interaction, intention to treat
[c] Prespecified time point, intention to treat
